# Pulmonary Vein Thrombosis: An Unlikely Cause of Chest Pain

**DOI:** 10.7759/cureus.74408

**Published:** 2024-11-25

**Authors:** Sonia I Vicenty-Rivera, Alex P Rodriguez

**Affiliations:** 1 Cardiology, Bruce W. Carter, Miami VA (Veterans Affairs) Healthcare System, Miami, USA; 2 Cardiology, VA (Veterans Affairs) Caribbean Healthcare System, San Juan, PRI; 3 Cardiology, Bruce Carter Miami VA (Veterans Affairs) Healthcare System, Florida, USA

**Keywords:** anticoagulation, doacs, doacs and pvt, pulmonary emboli, shortness of breath (sob), therapeutic anticoagulation

## Abstract

Pulmonary vein thrombosis (PVT) is a rare but potentially lethal source of arterial thromboembolism. We present a case of a 59-year-old female patient who presented with worsening shortness of breath and was found on a cardiac computed tomography angiography (CTA) with the right lower pulmonary vein partially occluded with a filling defect. This case report highlights the diagnostic challenges and management options associated with this rare entity.

## Introduction

Pulmonary vein thrombosis (PVT) is a rare but potentially life-threatening condition characterized by the formation of a thrombus within the pulmonary veins [1,2]. While deep venous thrombosis thromboembolism (VTE) predominantly affects the deep veins of the lower extremities, in a few cases, it has been described as thrombus formation at the level of the pulmonary venous circulation; hence, PVT has garnered attention due to its significant implications for respiratory and cardiovascular health [2]. The most frequently encountered cause for this diagnosis is typically related to the presence of intrapulmonary neoplasms, lung transplantation, and upper lobectomy surgeries [2-7]. Only a handful of cases described in the literature are primarily associated with early post-lung transplant or lobectomy surgery complications, primary or secondary lung cancers, or radiofrequency ablation for atrial fibrillation [3-8]. Other conditions associated with PVT may include complications of radiofrequency ablation, fibrosing mediastinitis, mitral stenosis with a left atrial clot, hilar torsion, hypercoagulable state, sickle cell trait/anemia, inflammatory bowel disease, tuberculosis, and Janus kinase inhibitors [7,8]. We present a case of a 58-year-old female without prior cardiovascular events who presented with symptoms of chest pain and was subsequently diagnosed with pulmonary vein thrombosis and none of the above predisposing conditions. This case highlights the pathophysiology, risk factors, clinical manifestations, diagnostic challenges, and treatment options associated with pulmonary vein thrombosis, emphasizing the need for heightened awareness among healthcare professionals.

## Case presentation

**Figure 1:**
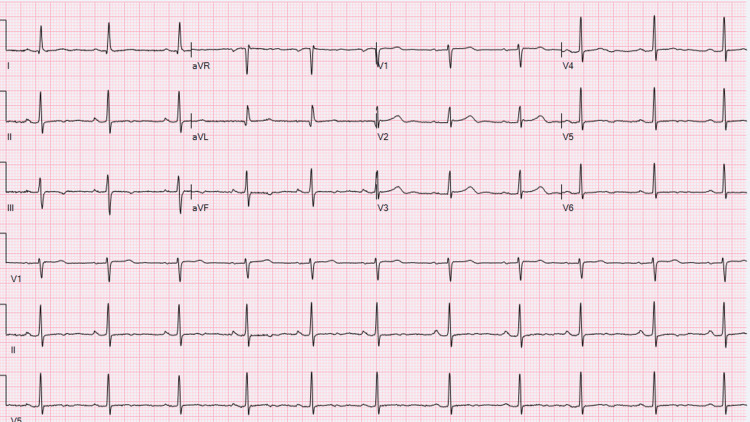
12-lead electrocardiogram performed for evaluation of chest pain with sinus rhythm with non-specific ST-T changes

A 57-year-old female patient (G1, P0, C1, A0) with arterial hypertension, diabetes mellitus, hypothyroidism, and dyslipidemia and former military personnel who served in Southeast Asia presented with chest pain localized on the right sternum area of moderate intensity. The pain was stabbing-like, lasting several minutes, and relieving on its own. The patient also reported occasional non-limiting, short-lasting palpitations. The patient denied lower extremity swelling, increased abdominal girth, paroxysmal nocturnal dyspnea, orthopnea, cough, fatigue, fevers, chills, night sweats, syncope, or near syncope. She reported COVID-19 viral illness with bronchitis five months before chest pain symptoms started. She denied any cough, sputum production, or hemoptysis after COVID-19 bronchitis resolved. A chest CT performed during the event only showed right lobe atelectasis, believed to be a manifestation of the viral illness, but no infiltrations; therefore, symptoms resolved with antiviral treatment and denied a family history of cardiovascular, rheumatological, or hematological conditions. The patient also denied a recent history of long travels, lung infections other than COVID-19, prothrombotic state, sickle cell disease, chest wall trauma, cardiac or valvular disease, or any other relevant history. The patient was exposed to burning pits of chemicals during her military service. Physical examination did not disclose any pathological findings such as rales, rhonchi, wheezes, extra heart sounds, and room air oxygen saturation of 98%. A 12-lead ECG showed normal sinus rhythm and nonspecific T wave changes (Figure 1).

**Figure 2:**
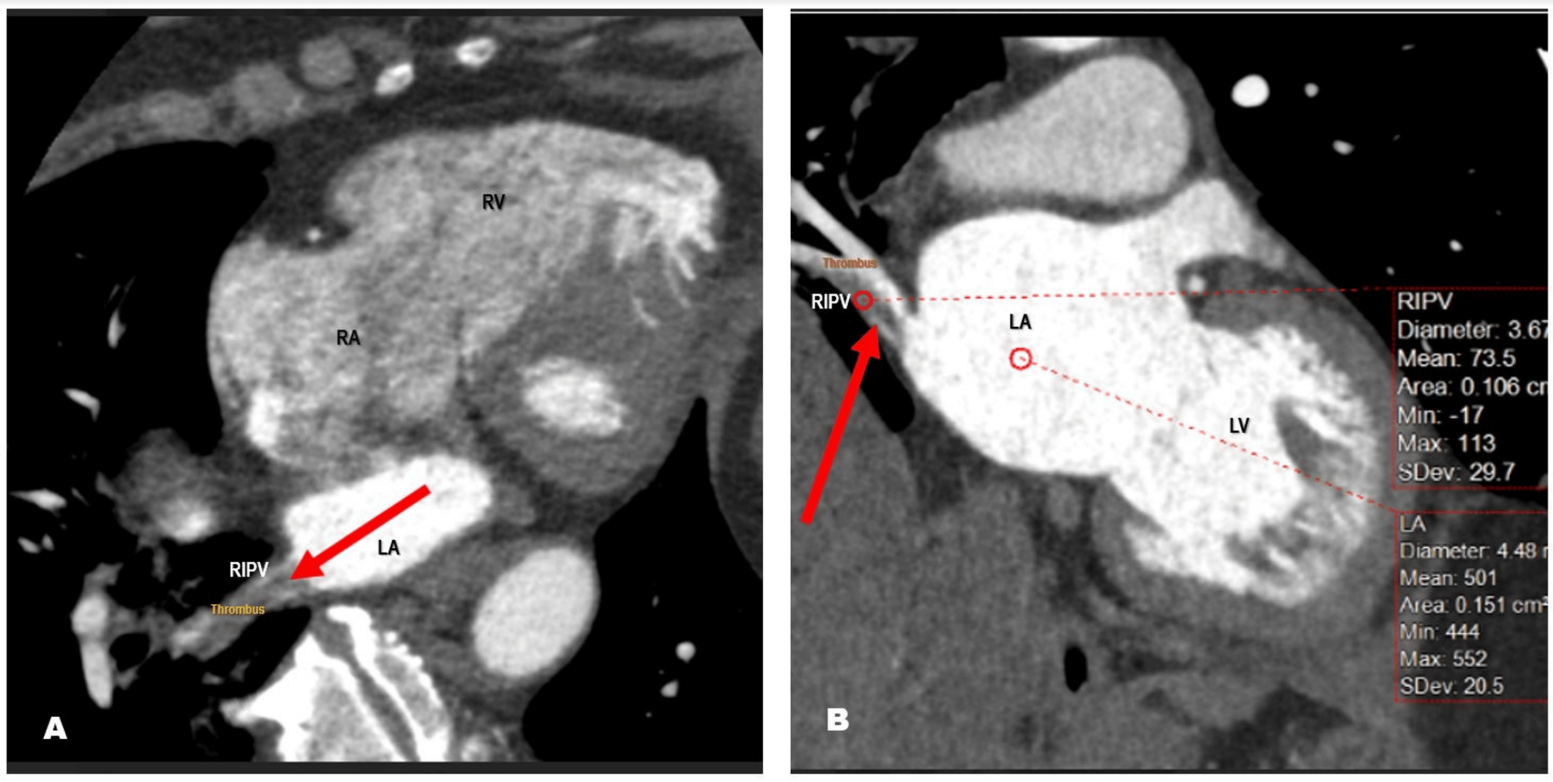
Coronary computer angiography A and B show the right inferior pulmonary vein is small in caliber, measuring 0.5 sq cm in area. The right lower pulmonary vein appears partially chronically occluded with a filling defect. Its branches correspond to the posterior basilar right lower lung.

Basic laboratories included CBC with differential, comprehensive metabolic panel and urinalysis were within normal limits without anemia, preserved renal parameters (estimated glomerular filtration rate (eGFR) 70), and no proteinuria. A coronary CTA was performed for the patient's symptoms and cardiac risk factors (10-year atherosclerotic cardiovascular disease (ASCVD) score of 10% and lifetime ASCVD score of 39%). The study showed non-obstructive coronary disease, but the right lower pulmonary vein appeared partially chronically occluded with a filling defect with a nonocclusive filling defect in the right inferior pulmonary vein, consistent with a thrombus, extending to the left atrial ostium (Figure 2).

**Figure 3:**
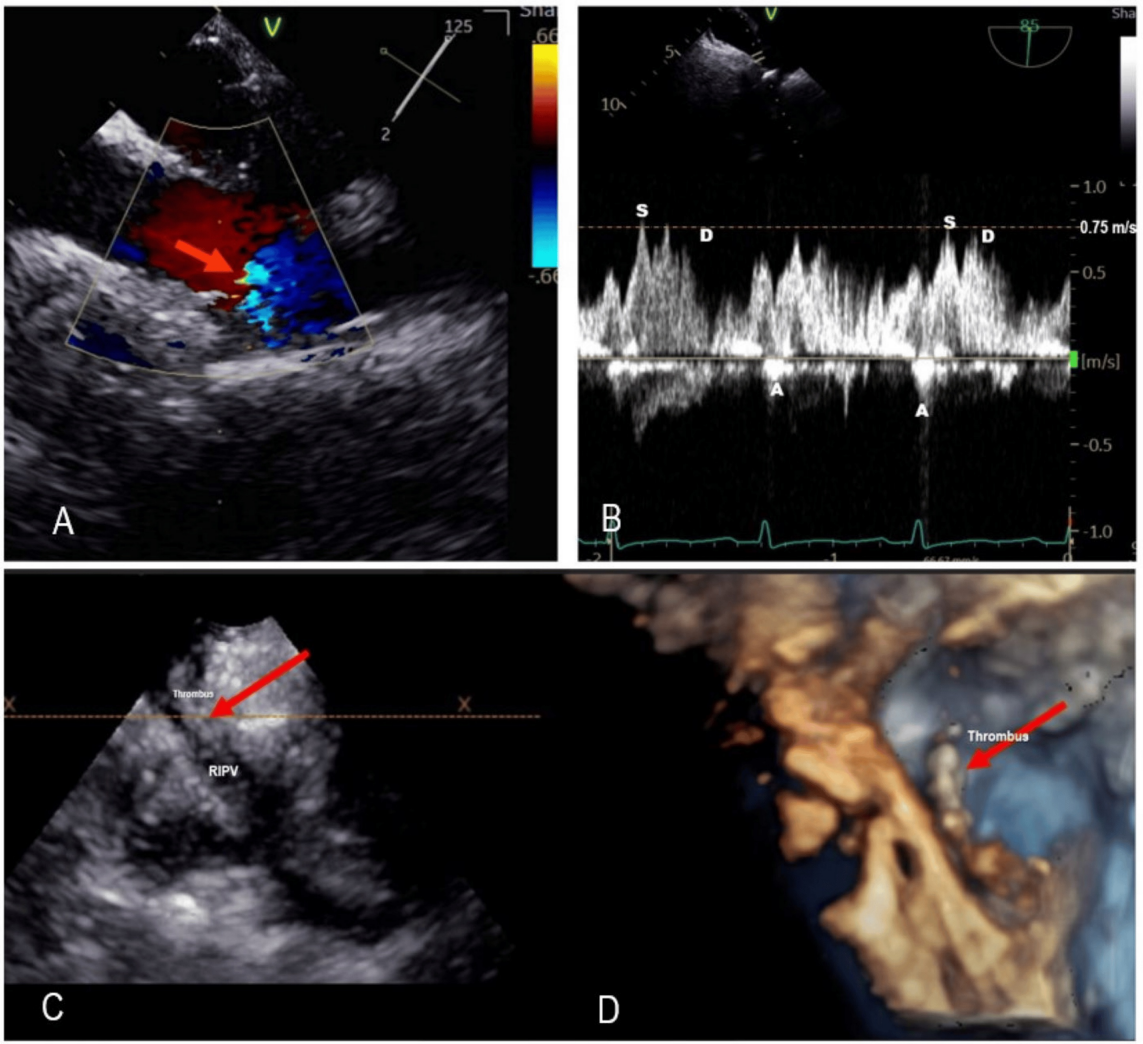
TEE 2D TEE of the RIPV performed at 45º-60º showed turbulent flow seen with CF-Doppler (4A) and PW-spectral Doppler has pulmonary vein S & D waves velocities at 0.75 m/s (4B); both findings are associated with pulmonary vein thrombosis and obstruction. 2C. 45º-60º shows the pulmonary vein had a small ostium caliber with an echogenic structure decreasing its lumen. The 3D TEE (2D) shows the echogenic structure is a thrombus. 2D TEE: two-dimensional transesophageal echocardiogram; 3D TEE: three-dimensional transesophageal echocardiogram; RIPV: right inferior pulmonary vein; PW-Doppler: pulse wave Doppler; CF-Doppler: color flow Doppler; S wave Velocity: pulmonary systolic vein flow; D wave velocity: pulmonary diastolic vein flow

A transesophageal echocardiogram (TEE) was performed to improve visualization and determine the thrombus's size (Figure 3C). The study showed normal systolic function (LV ejection fraction = >65%), a normal-sized left atrium, and average-sized superior and inferior left pulmonary veins. There is no evidence of a left atrial or left atrial appendage thrombus. The right atrium appears normal in size. The right superior pulmonary vein was normal. The right inferior pulmonary vein had a small ostium and caliber of the vessel. Doppler interrogation showed turbulent color flow-Doppler and decreased Pw-Doppler flow velocities (Figure 3B).

Specific laboratory tests for hereditary thrombophilia excluded inherited prothrombotic conditions (Table 1). The patient was prescribed direct oral anticoagulants (DOAC) to manage pulmonary vein thrombosis. Lung, gastrointestinal (GI), and genitourinary (GU) malignancies were excluded, and a chest, abdominal, and pelvic CT was performed to rule out the possibility that they might predispose to prothrombotic conditions.

**Table 1:** Laboratory workup for hereditary thrombophilia 1. Protein C Activity; Normal 70-180%; 2. Protein C Activity; Normal 60-140%; 3. Factor VIII Activity; Normal 80-120%

Hereditary thrombophilias	
Protein C Activity (%)	192
Protein S Activity (%)	151
Factor VIII Activity (%)	147
Antithrombin III Activity (%)	120
Factor V (Leiden) Mutation Analysis	Negative
Lupus Anticoagulant	Not detected
Cardiolipin Ab, IgA, IgG, IgM	Not detected
Prothrombin (Factor II) Mutation Analysis	Negative

**Figure 4:**
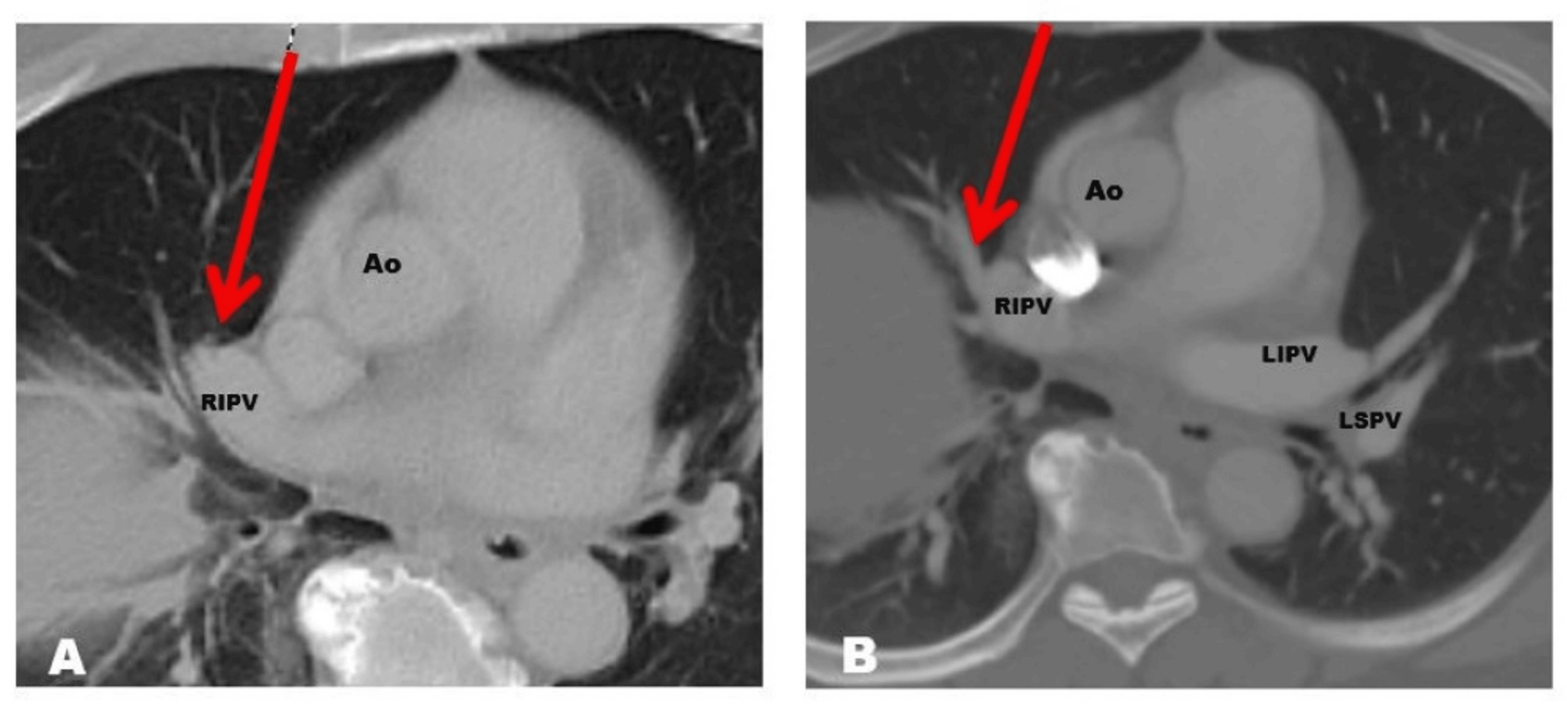
Chest CTA On the left (4A), dedicated chest CTA performed at baseline with occlusion of the RIPV (red arrow). On the right (4B), follow-up chest CTA performed at 12 months with DOAC treatment shows resolution of the RIPV thrombus. RIPV: right inferior pulmonary vein; CTA: computer angiography; DOAC: direct oral anticoagulant

The patient continued anticoagulation treatment with DOAC for 12 months without any complications or recurrence of events, after which time a follow-up Chest CT angiography was requested that showed complete resolution of thrombus (Figure 4). The study showed complete resolution of the thrombi; thus, anticoagulation treatment was discontinued without further complications.

## Discussion

Pulmonary vein thrombosis (PVT) is a rare condition characterized by the formation of blood clots within the pulmonary vein vasculature, which can migrate to the left atrium. However, specific clinical conditions can lead to the obstruction of the pulmonary veins and subsequent infarction [1]. Recently, it has been noted that severe acute respiratory syndrome coronavirus 2 (SARS-CoV-2) COVID-19 infection is also recognized as a risk factor for PVT due to the heightened hypercoagulable state during the acute infectious process. Nevertheless, the duration of the hypercoagulable state or its association with the long COVID phase is unknown. This may be the contributed etiology in our patient's diagnosis [7-9]. PVT occurs rarely due to the protective effect of a rich network of venous collateral vessels that drain the lung. Nevertheless, when it happens, it involves multiple factors, such as the combination of venous stasis, endothelial injury, and hypercoagulability, collectively described as Virchow's triad. In cases of primary or secondary malignancy, PVT is believed to develop as a result of the tumor's direct extension into the vein, leading to vein compression by the tumor as well as a hypercoagulable state with epithelial damage from tumor invasion. Additionally, in procedures such as lobectomy and lung transplant involving the pulmonary venous anastomotic site, thrombus formation can occur. In terms of comorbidities, diabetes mellitus (DM) is considered a risk factor for pulmonary embolism (PE), a potentially life-threatening consequence of venous thromboembolism (VTE); it has not been associated with pulmonary vein thrombosis [10].

In pulmonary embolism, pulmonary angiography is the gold standard for diagnosis. On the other hand, for PVT, no standard study is used to identify the disease, and various imaging techniques are required. PVT can be diagnosed incidentally in asymptomatic patients undergoing a chest CT angiography to evaluate unrelated clinical conditions [11]. When symptoms appear, they are non-specific and can range from cough, exertional shortness of breath, and chest pain to more severe symptoms such as hemoptysis, lung edema, fever, and infarction with or without hypoxemia [12,13]. The potential consequences of PVT can be severe, leading to respiratory failure, stroke, systemic embolization, and even death. Chest radiography does not help diagnose this condition, as it often shows non-specific changes such as lobar consolidation and pleural effusions [7]. Therefore, a multi-imaging approach is frequently required, using techniques such as chest CT scanning, transesophageal echocardiography, magnetic resonance imaging, and pulmonary angiography.

A chest CT scan using a 64-slice multidetector computed tomography (64-MDCT) can detect pulmonary vein stenosis or obstruction and provide information about the underlying causes of PVT [14]. It can reveal signs of interlobular septal thickening and ground-glass opacification caused by localized pulmonary edema, enlarged lymph nodes, lymphangiectasis, and pleural effusion due to impaired venous drainage. It can also show venous collateral formation, thickening of broncho-vascular bundles due to ectasia, and remodeling of the bronchial arteries and veins. Additionally, it may reveal pleural plaques due to the organization of fibrinous exudates and visible interstitial fibrosis due to the accumulation of hemosiderin-containing macrophages in the interstitial space. It is essential to consider pulmonary vein artifacts such as pulmonary vein smoke within the differential diagnosis. It can be better evaluated with other cardiac imaging studies such as a transesophageal echocardiogram (TEE).

The use of TEE helps in visualizing and ruling out the presence of blood clots in the pulmonary veins, determining the size of pulmonary vein clots, assessing the diameter of the remaining pulmonary vein, and examining the extension into more prominent pulmonary veins and the left atrium. TEE also aids in measuring the velocity of blood flow in the pulmonary veins, which can indirectly help diagnose PVT. A normal spectral Doppler pulmonary vein tracing typically displays low-velocity waves, including the systolic (S) wave, diastolic (D) wave, and atrial reversal (AR) wave, with expected velocities for the S and D waves falling within the range of 30-60 cm/s [15]. An increase in spectral Doppler S and D velocities to >100 cm/s, along with a reversal of S to D wave predominance (S<D) and turbulent flow seen on color flow Doppler, strongly suggests a narrowed pulmonary vein segment or pulmonary vein thrombosis [16,17]. Furthermore, MRI techniques can be utilized for both screening and diagnosing PVT. It benefits patients who cannot use iodinated contrast medium due to allergic reactions or renal dysfunction. Chest magnetic resonance imaging is valuable for distinguishing between bland thrombus formation and a tumor or tumor/thrombus within the pulmonary vein [7].

PVT management largely depends on its underlying cause, and anticoagulation is the primary treatment. Unlike PE or other venous thromboembolism (VTE), where direct oral anticoagulants (DOACs) are the preferred treatment, current guidelines do not specify the type of anticoagulation or the duration of therapy for patients with PVT [18,19]. Most knowledge about PVT management is based on individual cases and small, primarily retrospective studies. Asymptomatic PVT, as in our patient, generally does not have clinical consequences and does not require treatment changes. However, complications can occur if left untreated, including pulmonary infarction, pulmonary edema, right ventricular failure, allograft failure, peripheral embolism, limb ischemia, stroke, and renal infarction [7]. Although less commonly reported, peripheral embolism can occur and has resulted in limb ischemia, stroke, and renal infarction [7]. Sykora et al. published a small study in 2024 in which 72 patients were diagnosed with PVT and were followed during a mean period of 14 months [19]. During that period, complications such as left atrial thrombus (21%), need for mechanical ventilation (14%), pneumonia (9%), and ischemic stroke (9%) were seen in that population. Furthermore, this study suggested a mortality rate of 46%, with a median survival of 14 months after PVT diagnosis. Much of this mortality was driven likely due to underlying conditions such as cancer [20].

## Conclusions

Pulmonary vein thrombosis remains an underrecognized but critical entity within the spectrum of venous thromboembolism. Its multifactorial etiology, diverse clinical manifestations, and potential for severe complications necessitate a proactive approach to diagnosis and management. Increased awareness among healthcare providers and a thorough understanding of risk factors and treatment options are essential for improving patient outcomes. Future research should focus on elucidating the pathophysiology of PVT, refining diagnostic techniques, and developing targeted therapeutic strategies to mitigate the impact of this condition on public health. As the medical community advances in understanding and treating PVT, patients can expect better recognition and management of this complex disorder.
